# Supplementary material for: Identification and Characterization of Static Craniofacial Defects in Pre-Metamorphic Xenopus laevis Tadpoles
Source: J Dev Biol. 2025 Jul 25;13(3):26. doi: 10.3390/jdb13030026 (PMC12371963; doi:10.3390/jdb13030026)
Supplement: Supplementary file 1 [file jdb-13-00026-s001.zip › jdb-3557942-supplementary.pdf]

Supplementary Materials

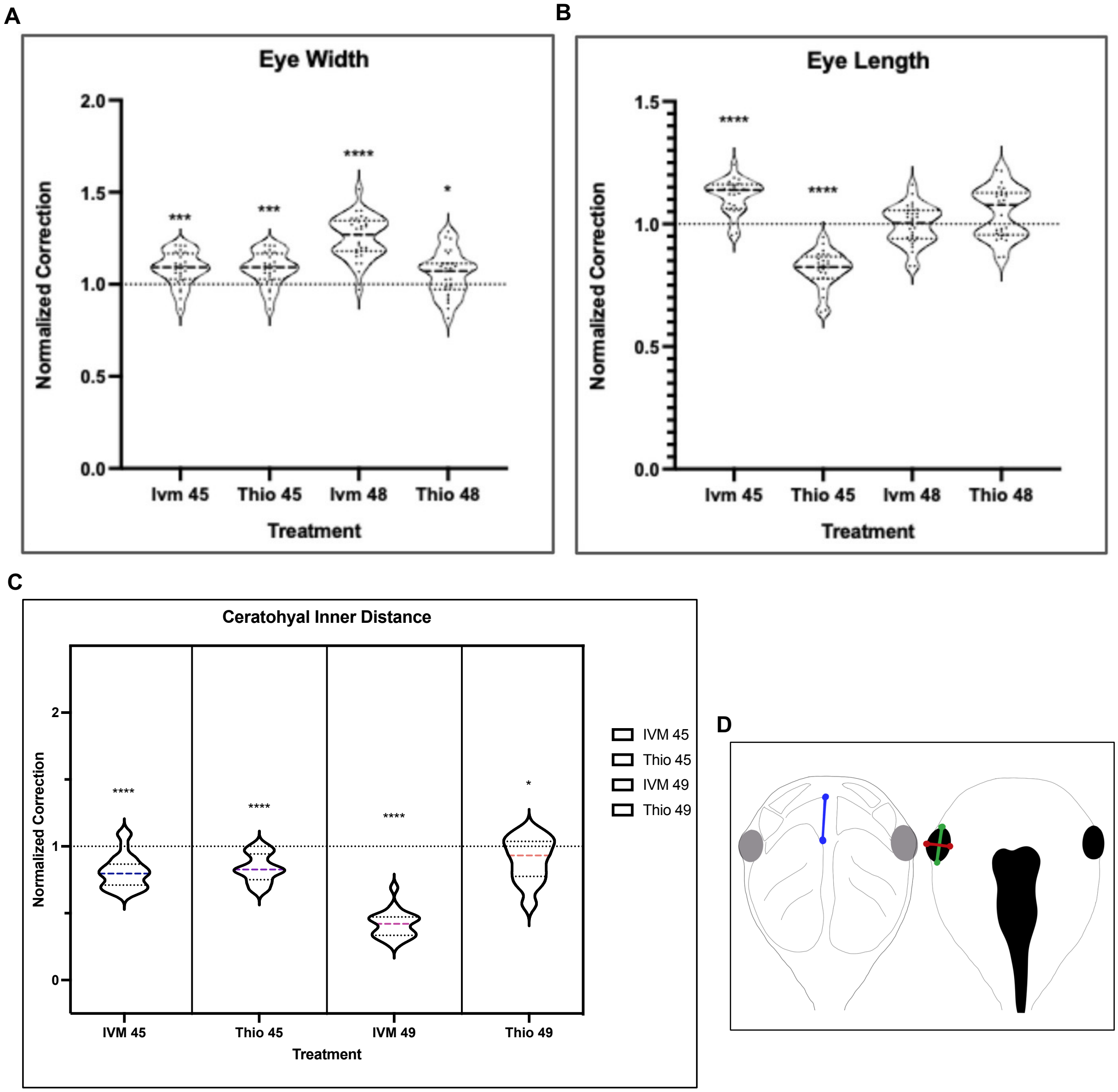

Figure S1. Additional Morphometric Landmark Analysis. Morphological landmarks assessments for [A] eye width (lateral/medial spread), [B] eye length (rostral/caudal spread), and [C] ceratohyal rostral caudal lengthening. Treated tadpoles were normalized to staged matched untreated controls at stages 45 and 49. A schematic of geometric landmarking is shown in panel D (Ceratohyal length in blue, eye length in green, and eye width in red). A two-tailed Mann-Whitney U-test was used for analysis. ns=P>0.05, *P<0.05, **P<0.01, ***P<0.001, ****P<0.0001. An N of 1 is represented for each landmark with 24 to 30 animals used for each treatment group.

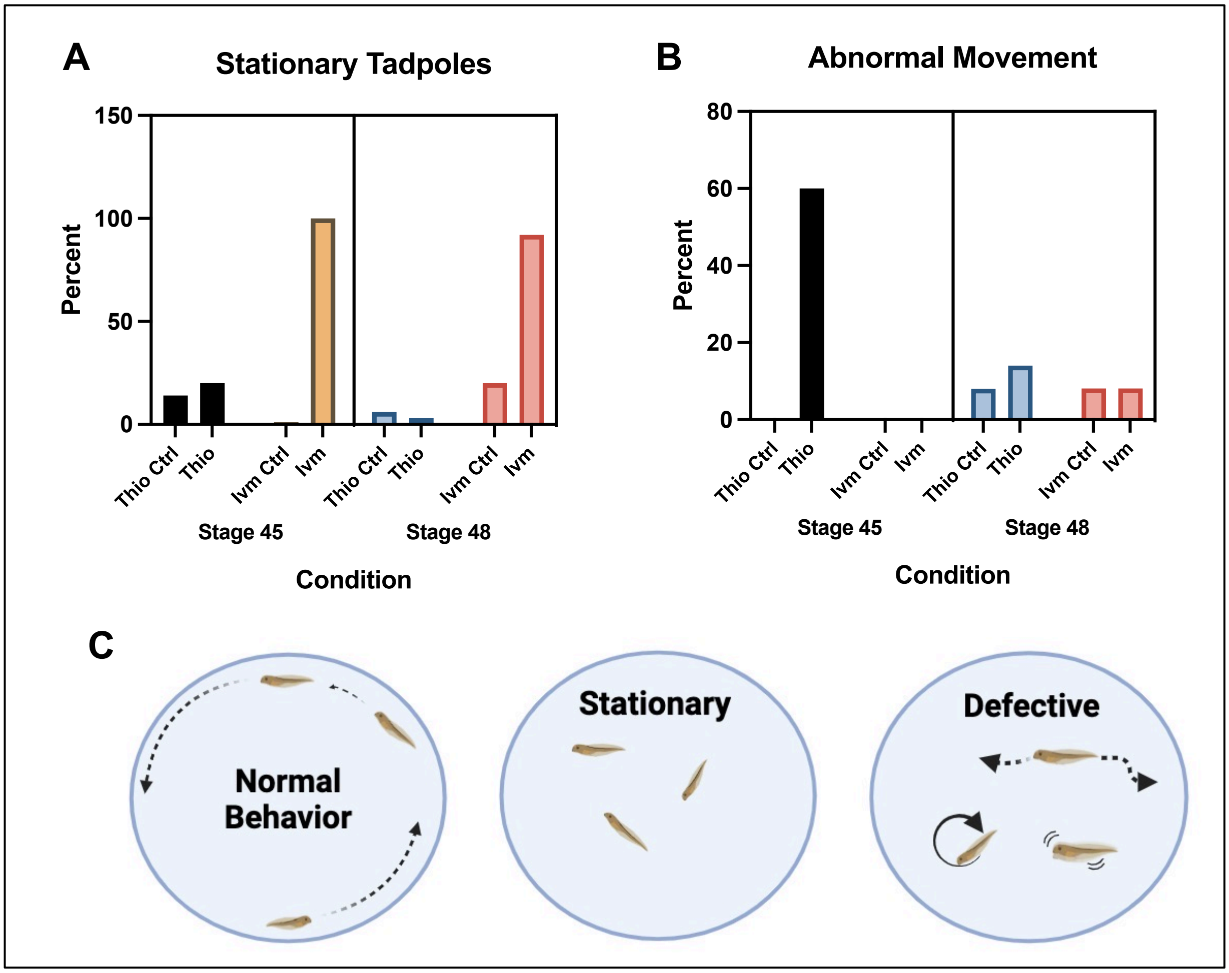

Figure S2. Quantification of Behavior. Behaviors were quantified from videos taken at stages 45 and 48 with a full lack of movement categorized as stationary and any non-coordinated or directional movement categorized as abnormal. Examples of abnormal behaviors include twitching, spinning, and frequent direction changes while moving. Graphics of these behaviors seen in panel C. Ivermectin and thioridazine videos were captured from separate fertilizations and thus are compared to their own individual control groups. Thio stage 45 and 48 videos are independent experiments due to lethality. (Ivm 45+48 and Ivm Cntrl 45+48: n=12, Thio 45: n=10, Thio Cntrl 45: n=35, Thio 48 and Thio Cntrl 48: n= 35).

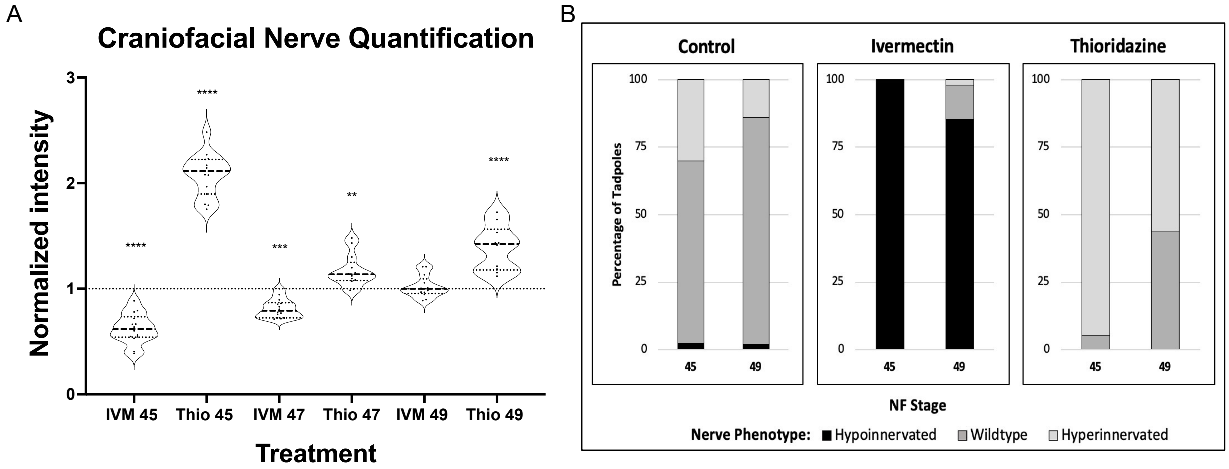

Figure S3. Nerve Quantification. [A] Quantification of florescent acetylated tubulin stain intensity at stages 45, 47, and 49 using ImageJ analysis [B] Tadpoles blind scored for hyper or hypo innervated nerve phenotypes as defined by increased branching vs reduced or missing nerves. N=2, n=32-45. Two-tailed Mann-Whitney U-test was used for analysis. ns=P>0.05, *P<0.05, **P<0.01, ***P<0.001, ****P<0.0001.

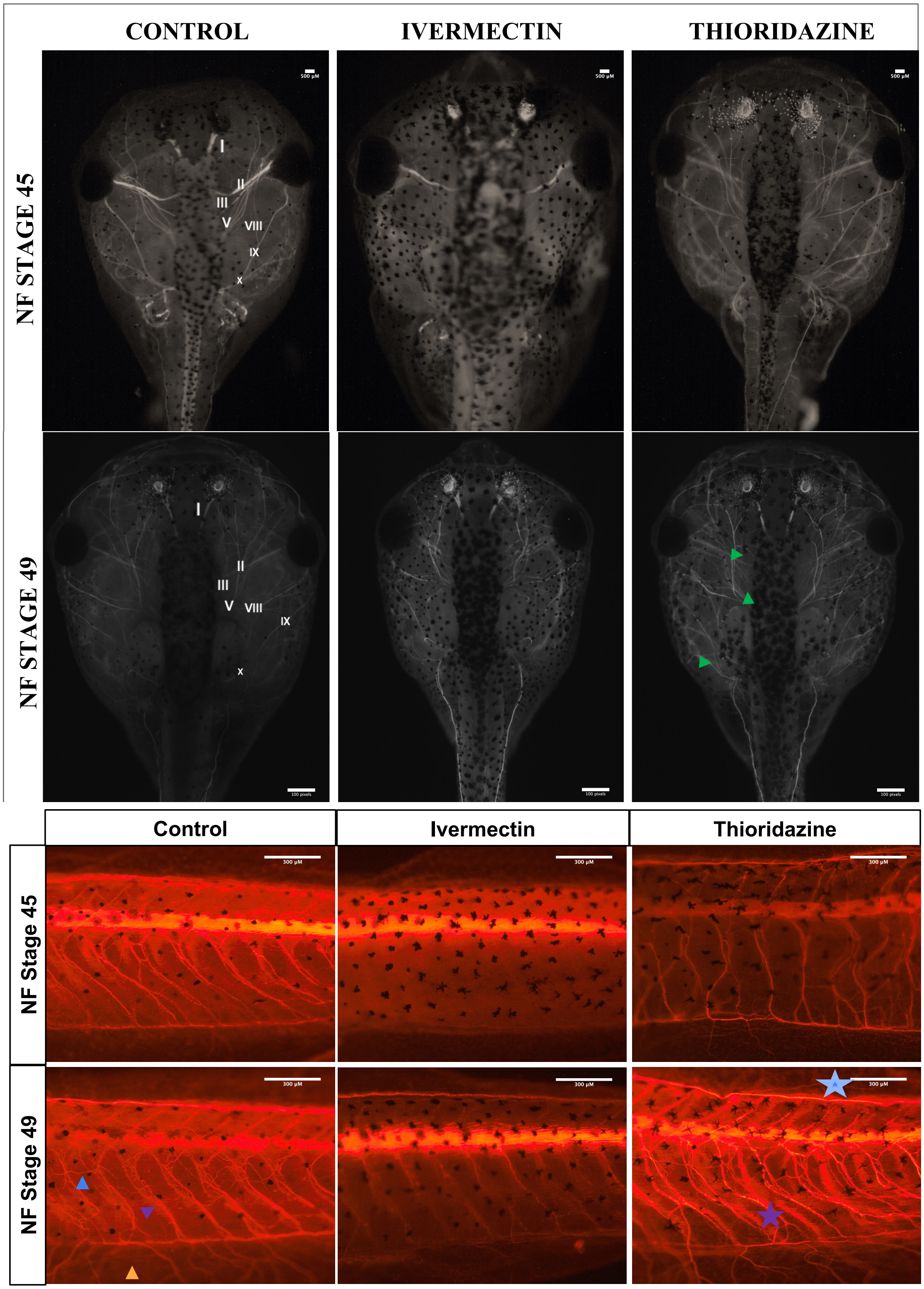

Figure S4. Unaltered Nerve Staining. Representative raw images with unadjusted brightness or contrast used for quantitative analysis.

Table S1. RT-qPCR Primer Sequences. Forward and reverse primer sequences and their corresponding gene IDs.

**Gene**	**Gene Symbol**	**NCBI Gene ID**	**Forward and reverse sequences (5′-3′)**
prl.2	*prl.2.S*	108697263	F: CCGTCAGATTTTAGGGAAAGCC<br>R: CTGGTTCCATGAGCGCAGTA
thrα	*thra.L*	397942	F: AGAAGCTGCCCATGTTCTCT<br>R: ACCTCCGTTCTTAAGCTGCT
thrβ	*thrb.L*	779054	F: CATAGTTAATGCGCCCGAGG<br>R: TGTCACTGCCATCTCACCAT
eef1a	*eef1a1*	1915	F: TGGATATGCCCCTGTGTTGGATT<br>R: TCCACGCACATTGGCTTTCCT
mmp1	*mmp1.S*	495287	F: AAAGAATTGATGCGGCTGTTCA<br>R: GAGCTTGGGGTCCGTCTTATT
mmp13	*mmp13.S*	379564	F: TCCTCCAGACGAGCAGACAT<br>R: CATGGGCAGCAACAAGGAAC
